# Supplementary material for: Transcriptomic Insights: Phytogenic Modulation of Buffel Grass (Cenchrus ciliaris) Seedling Emergence
Source: Plants (Basel). 2024 Apr 23;13(9):1174. doi: 10.3390/plants13091174 (PMC11085557; doi:10.3390/plants13091174)
Supplement: Supplementary file 1 [file plants-13-01174-s001.zip › plants-2913295-supplementary.pdf]

Supplementary Table S1. Pathways and their related differentially expressed genes identified in PHY.

Pathway	Upregulated genes	Gene name (protein name)	Downregulated genes	Gene name (protein name)
**5-Hydroxytryptamine biosynthesis**	Os10g0400500	*OsTDC1*		
	Os07g0437500	*TDC2*		
**Chorismate biosynthesis**	Os09g0539100	3-dehydroquinate synthase		
	Os01g0102600	Shikimate kinase		
	Os10g0577700	Shikimate kinase		
**Cytoskeletal regulation by Rho GTPase**	Os10g0510000	*ACT2*	Os03g0836000	*ACT3*
	Os10g0323600	Profilin-A	Os12g0163700	-
	Os03g0718100	*Actin-1*		
	Os01g0282800	*TUBB1*		
	Os03g0661300	*TUBB8*		
**De novo purine biosynthesis**	Os02g0778400	UMP-CMP kinase 4	Os08g0500900	Phosphoribosylglycinamide formyl transferase 1
	Os03g0699300	*PURA2*	Os05g0270800	Phosphoribosylglycinamide formyl transferase 1
	Os03g0320900	*V2*		
	Os12g0236400	*ADK-A*		
	Os12g0548300	Nucleoside diphosphate kinase		
	Os03g0102100	Formyltetrahydrofolate deformylase family protein		
	Os08g19140	Adenylate monophosphate kinase 5		
**Formyltetrahydrofolate biosynthesis**	Os10g0503200	Folylpolyglutamate synthase		
	Os03g0102100	Formyltetrahydrofolate deformylase family protein		
**Heme biosynthesis**	Os03g0337600	Uroporphyrinogen decarboxylase 2	Os01g0631200	Uroporphyrinogen-III C-methyltransferase
	Os03g0340001	-		
	Os02g0168800	*HEMC*		
	Os02g0121000	Glutamate--tRNA ligase		
	Os01g0622300	Uroporphyrinogen decarboxylase 1		
	Os06g0704600	*HEMB*		
	Os08g0532200	*GSA*		
**Isoleucine biosynthesis**	Os05g0558400	Branched-chain-amino-acid aminotransferase		
	Os02g0510200	*ALS1*		
**Leucine biosynthesis**	Os05g0558400	Branched-chain-amino-acid aminotransferase		
	Os03g0655700	3-isopropylmalate dehydrogenase		
**Lysine biosynthesis**	Os03g0118600	Dihydrodipicolinate reductase, C-terminus family protein	Os07g0461900	-
	Os03g0850700	-	Os05g0129100	-
	Os02g0436400	*DAPB1*		
	Os03g0117000	-		
	Os07g0300900	Aspartate kinase		

**Pyruvate metabolism**	Os08g0562100	Malate dehydrogenase (NADP(+))	Os02g0232400	Citrate synthase
	Os02g0194100	Citrate synthase	Os12g0183100	2-oxoisovalerate dehydrogenase alpha subunit
	Os01g0300200	*ACLB-1*		
	Os02g0739600	Pyruvate dehydrogenase E1 component subunit alpha-1		
**Salvage pyrimidine ribonucleotides**	Os05g0455700	Uracil phosphoribosyl transferase		
	Os02g0778400	UMP-CMP kinase 4		
	Os05g0182201	Hypoxanthine phosphoribosyl transferase		
**TCA cycle**	Os08g0562100	Malate dehydrogenase (NADP(+))		
	Os02g0194100	Citrate synthase		
	Os02g0739600	Pyruvate dehydrogenase E1 component subunit alpha-1(PDHE1-A)		
	Os07g0577700	Succinate--CoA ligase [ADP-forming] subunit alpha		

Supplementary Table S2. Pathways and their related differentially expressed genes identified in PHY.

Pathway	Upregulated genes	Gene name (protein name)	Downregulated genes	Gene name (protein name)
**5-Hydroxytryptamine degradation**	Os01g0591000	*ALDH1a*	Os02g0730000	*Aldh2a*
			Os06g0270900	*Aldh2b*
			Os09g0440300	Aldehyde dehydrogenase (NAD(+))
**Apoptosis signalling pathway**	Os01g0908100	*MPK3*	Os02g0148100	*MPK3*
	Os08g0157000	*MPK2*	Os03g0276500	70 kDa heat shock protein
	Os02g0797400	*MCM5*	Os11g0703900	Heat shock cognate 70 kDa protein
	Os01g0885000	CYTOCHROME C-2	Os06g0699400	*MPK4*
	Os05g0420600	*Cytochrome c*	Os03g0710500	*BIP2*
**Cadherin signalling pathway**	Os10g0510000	*Actin-2*	Os03g0836000	*ACT3*
	Os03g0718100	*Actin-1*	Os12g0163700	-
**De novo pyrmidine ribonucleotides biosythesis**	Os02g0778400	UMP-CMP kinase 4	Os01g0616900	CTP synthase
	Os12g0548300	Nucleoside diphosphate kinase		
**Folate biosynthesis**	Os10g0503200	Folylpolyglutamate synthase	Os08g0500900	Phosphoribosylglycinamide formyl transferase 1
	Os03g0102100	Formyltetrahydrofolate deformylase family protein	Os05g0270800	Phosphoribosylglycinamide formyl transferase 1
**Glycolysis**	Os07g0181000	*OsPK2*	Os08g0478800	Glucose-6-phosphate isomerase
	Os09g0535000	Triosephosphate isomerase	Os09g0465600	Glucose-6-phosphate isomerase
**Pentose phosphate pathway**	Os08g0154300	Transaldolase	Os08g0478800	Glucose-6-phosphate isomerase
			Os06g0129100	-
			Os09g0465600	Glucose-6-phosphate isomerase
**Threonine biosynthesis**	Os03g0850700	-	Os01g0693800	Threonine synthase
	Os07g0300900	Aspartate kinase		
**Tryptophan biosynthesis**	Os04g0467700	Indole-3-glycerol-phosphate synthase	Os06g0632200	Tryptophan synthase
			Os08g0135900	Tryptophan synthase

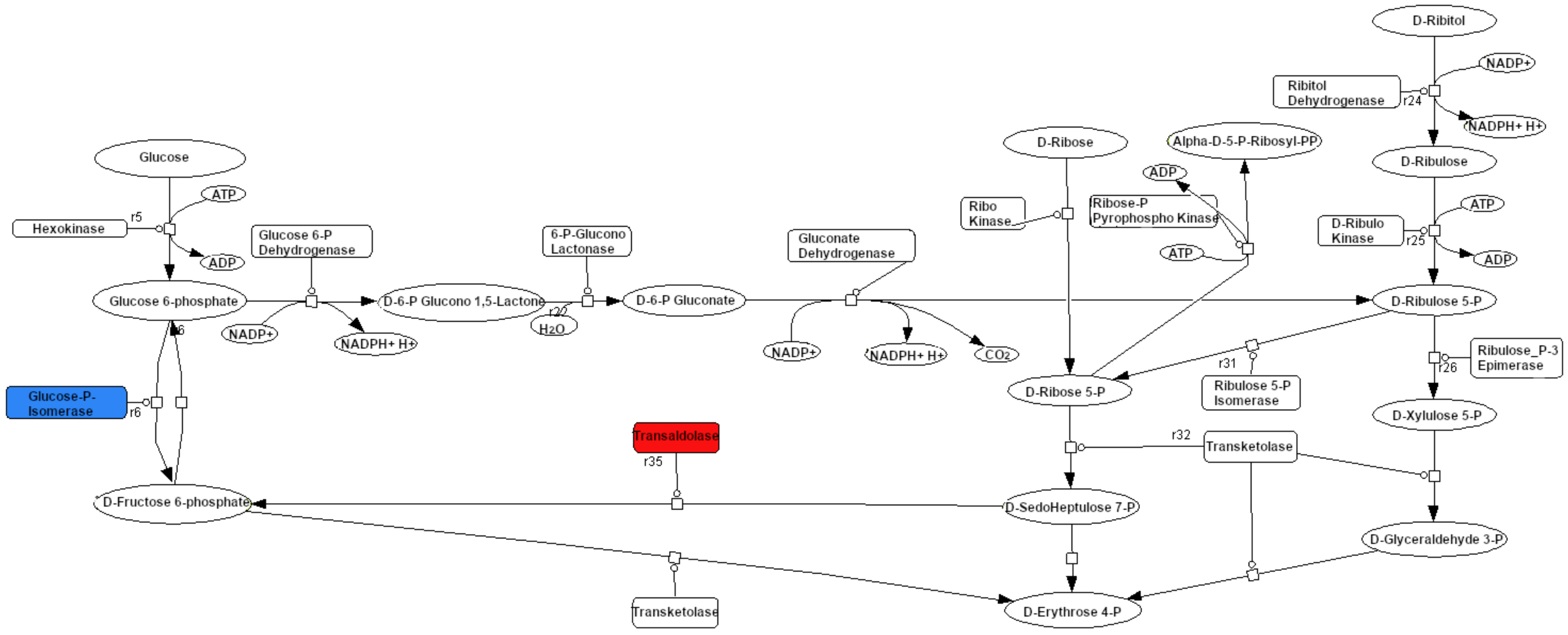

Supplementary Figure S1. Pentose phosphate pathway. The regulated enzymes in the control and treatment are highlighted by blue and red, respectively.

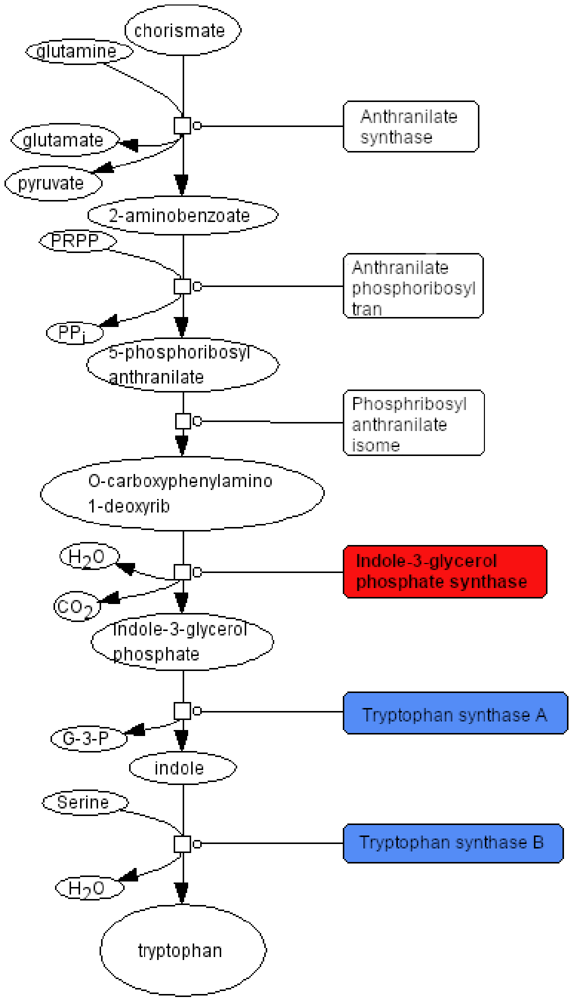

Supplementary Figure S2. Tryptophan biosynthesis with differential expressed genes in PHY. The inhibitory and activated enzymes are highlighted by blue and red, respectively.

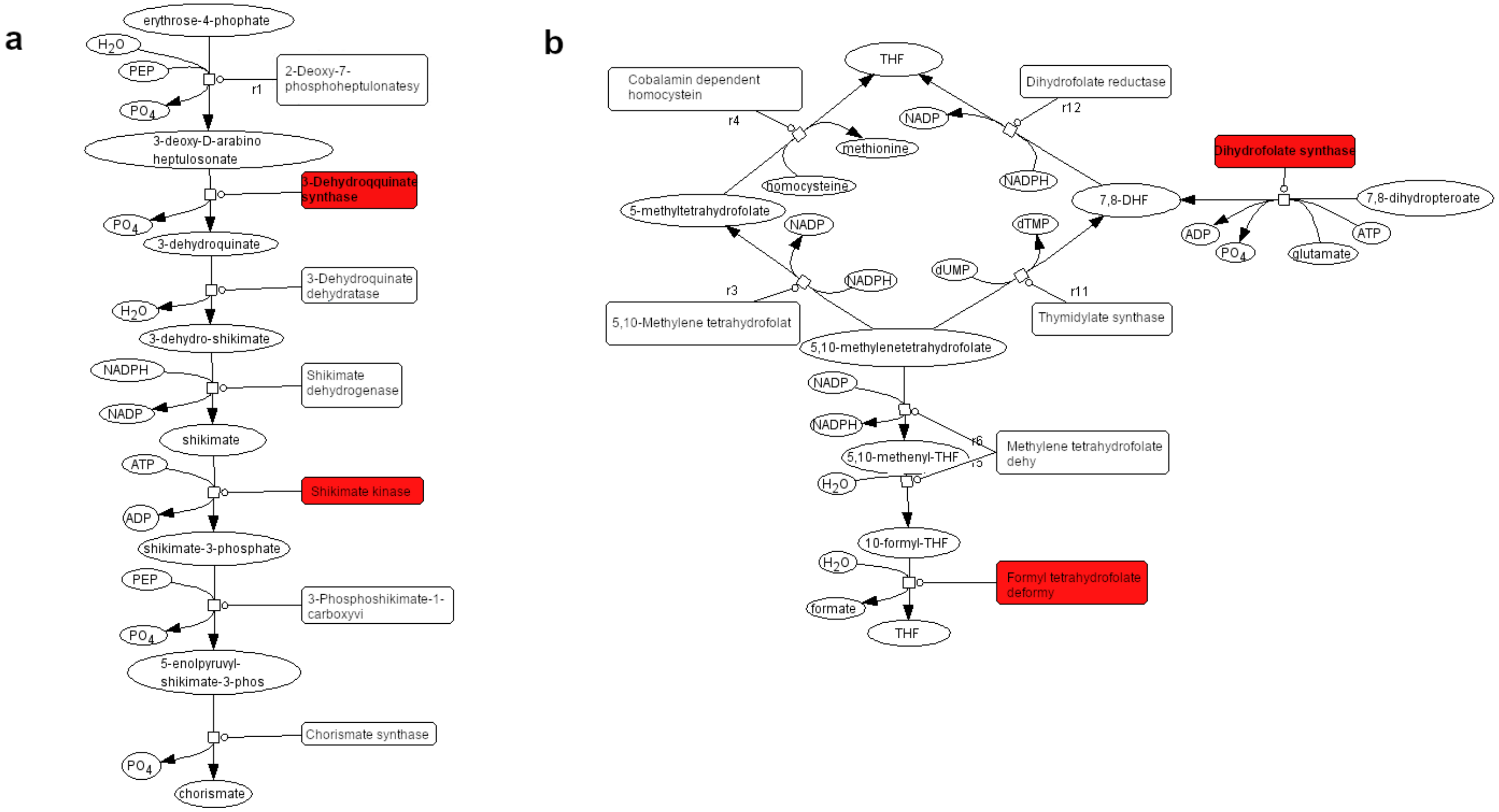

Supplementary Figure S3. Pathways with differential expressed genes in PHY. Panel a) represents chorismate biosynthesis; panel b) represents formyltetrahydrofolate biosynthesis. Enzyme encoded by highly expressed gene is highlighted by red.

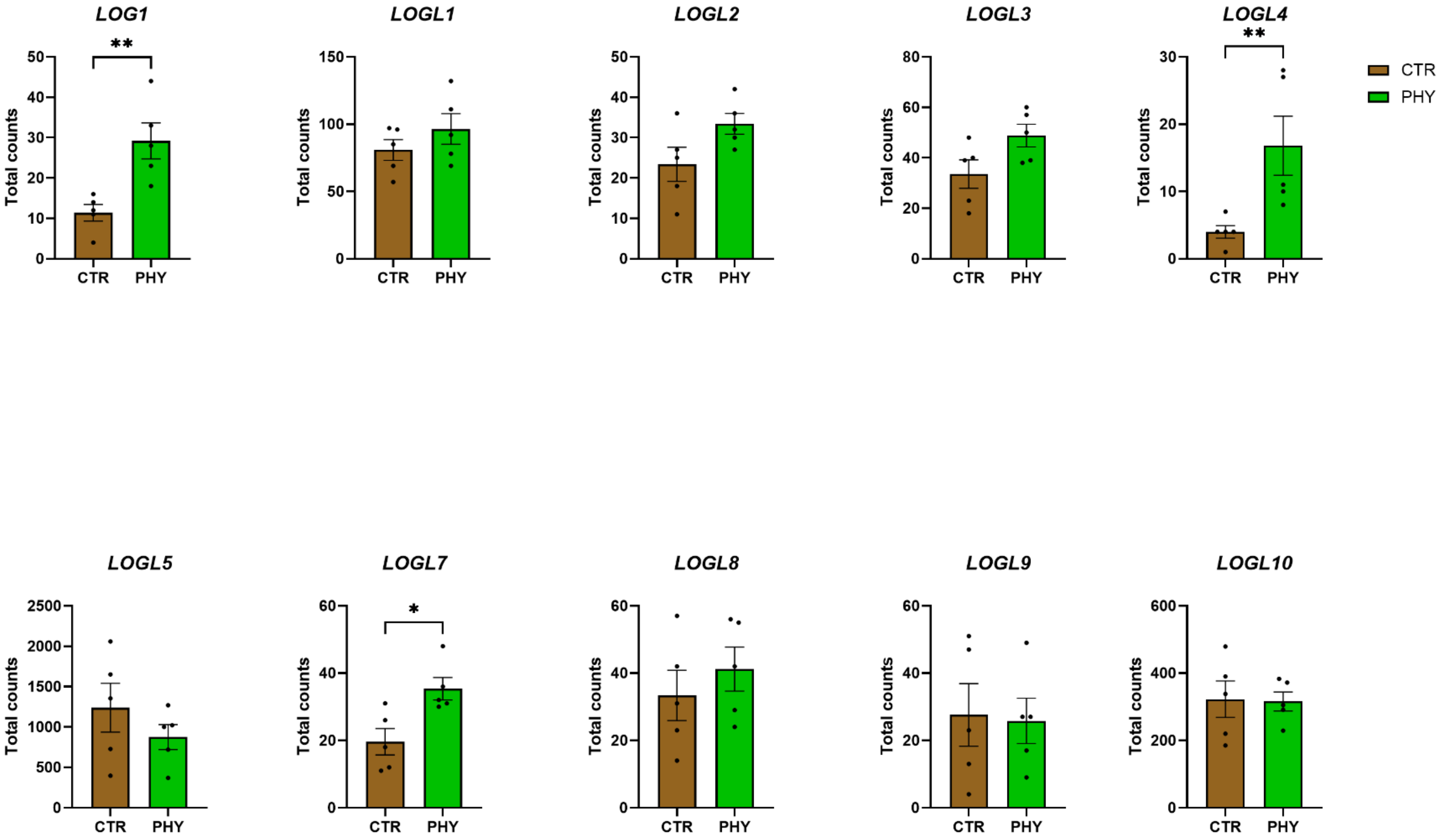

Supplementary Figure S4. *LOG* gene family RNA-seq count between control and PHY treatment. Data are mean ± SEM (n = 5). Asterisks indicate significant difference by Mann-Whitney t-test, * = $p < 0.05$, ** = $p < 0.01$.
